# Accuracy of frameless stereotactic brain biopsy: a retrospective cohort study with MRI-only and MRI-CT fusion navigation

**DOI:** 10.1007/s00701-025-06720-3

**Published:** 2025-12-20

**Authors:** Franziska Meinert, Patrick Dömer, Simeon O. A. Helgers, Levent Tanrikulu, Johannes Woitzik, Nikhil Wendt-Thakur

**Affiliations:** 1 Department of Neurosurgery, Carl Von Ossietzky University Oldenburg, Oldenburg, Germany; 2 Research Center Neurosensory Science, Carl Von Ossietzky University Oldenburg, Oldenburg, Germany; 3 Department of Neurosurgery, Jena University Hospital, Friedrich-Schiller-University, Jena, Germany; 4 Department of Neurosurgery, Evangelisches Krankenhaus Oldenburg, 26122 Oldenburg, Lower Saxony Germany

**Keywords:** CT-MRI Fusion, Frameless Biopsy, Intracranial Tumours, Neuronavigation, Neurosurgical Neurooncology, Targeting Accuracy

## Abstract

**Purpose:**

Stereotactic-guided biopsy remains the gold standard for diagnosing intracranial lesions not amenable to surgical resection. Frameless techniques, such as the VarioGuide® system (Brainlab AG, Munich, Germany), offer a minimally invasive alternative, typically using MRI-based navigation. However, MRI-based navigation may be affected by geometric distortions that impair targeting precision. CT imaging provides superior geometric fidelity. This retrospective analysis evaluates the accuracy of frameless stereotactic biopsies in clinical routine. Patients were grouped based on the imaging modality used for neuronavigation—either MRI-only or MRI-CT fusion—allowing secondary comparison between both approaches.

**Methods:**

In this retrospective cohort study, 99 patients who underwent frameless stereotactic biopsy between February 2022 and September 2024 were analysed. Patients were grouped by neuronavigation modality: CT-MRI fusion–based (*n* = 18) and MRI-only (*n* = 81). Accuracy was assessed by measuring entry and target deviations using postoperative CT. Lesion volume, depth, procedure duration, and complication rates were also evaluated.

**Results:**

Entry and targeting accuracy was comparable between groups (entry deviation: 5.2 ± 3.9 mm vs. 5.4 ± 3.0 mm, *p* = 0.84; target deviation: 4.2 ± 3.0 mm vs. 4.4 ± 2.7 mm, *p* = 0.85). Lesion volume and target depth showed no significant differences. No statistically significant differences in complication rates were observed between groups (27.8% vs. 11.1%, *p* = 0.14).

**Conclusion:**

MRI-only and CT-MRI fusion–based frameless stereotactic biopsies showed no statistically significant difference in targeting accuracy. While CT-based registration may theoretically reduce distortion-related errors, this was not reflected in our data. The choice of imaging modality should therefore be guided by clinical context and imaging availability. Further prospective studies are needed to clarify the value of CT integration in specific clinical scenarios.

## Introduction

For patients with unclear intracranial lesions, histopathological confirmation remains crucial for treatment planning, particularly when resection is not indicated. Stereotactic-guided biopsy remains the diagnostic gold standard, as clinical and radiological assessment alone proves insufficient in up to one-third of cases [9, 10, 13, 21].

Frameless systems, such as the VarioGuide® system (Brainlab AG, Munich, Germany), have become established alternatives to frame-based techniques, offering minimally invasive access with reduced operative time and favourable morbidity profiles [5, 6, 13, 15].

Studies confirm comparable accuracy and complication rates between both approaches, making the choice of technique dependent on lesion characteristics, surgeon experience, and institutional infrastructure [5, 11, 13, 19]. Lesion volume and biopsy trajectory are among the most important factors influencing diagnostic yield [5, 6, 11, 19, 21].

Frameless biopsies typically rely on MRI-based navigation. However, MRI guidance may be affected by geometric distortions—particularly in T2-weighted sequences—caused by susceptibility effects, gradient nonlinearity, and B₀ field inhomogeneities, especially near tissue–air or bone interfaces [17, 18, 22]. These distortions can be relevant in stereotactic procedures where submillimetre accuracy is essential.

To mitigate such limitations, image fusion with CT—which provides superior geometric fidelity—has been proposed [1, 11, 12]. CT-based navigation has also proven useful in other neurosurgical applications, such as electrode placement in deep brain stimulation [2, 7]. Recent studies using intraoperative CT platforms, such as mobile iCT units or the O-Arm, have reported promising results with acceptable accuracy and complication profiles [2, 4].

Although these prior reports illustrate the technical feasibility and high precision of MRI-CT fusion-based navigation, direct comparisons with MRI-only guidance—particularly in the context of frameless stereotactic biopsies for oncological indications—are still limited. Most available data focus on specific applications such as functional neurosurgery or small patient series, rather than systematic evaluations in broader clinical cohorts [1, 3, 8].

In this study, we retrospectively analysed the accuracy and safety of frameless stereotactic biopsies performed at our institution. Given that MRI-only and MRI-CT fusion navigation were both used during the study period depending on clinical and logistical considerations, we also explored differences between these two groups.

## Methods

### Patient recruitment

This retrospective study initially identified 113 patients who underwent frameless stereotactic biopsy for an intracranial lesion between 1 February 2023 and 9 September 2024. Patients were excluded if the procedure was converted to a frame-based approach due to technical difficulties, if a non-tumour-related intervention was performed, if no postoperative CT was available for analysis, if the trajectory used for biopsy was not digitally saved and thus unavailable for postoperative evaluation, or if the patient was under 18 years of age. Patients who underwent frameless stereotactic procedures for non-neoplastic conditions, such as abscesses or demyelinating lesions, were also excluded. As the study specifically aimed to analyse targeting accuracy and workflow in tumour biopsies, inclusion of non-tumorous cases could have introduced confounding due to differing tissue characteristics and radiological properties. Following these criteria, 99 patients were included in the final analysis.

All biopsies were performed using a frameless navigation system (VarioGuide®, Brainlab AG, Munich, Germany) Patients were retrospectively divided into two groups based on the imaging modality used for registration and navigation: (1) MRI-only, and (2) MRI–CT fusion, in which high-resolution cranial CT scans were fused with MRI data. Group allocation was not randomised. MRI-CT fusion navigation was applied when recent high-resolution CT data were available or when MRI image quality was limited by motion artefacts, susceptibility effects, or poor contrast resolution. In other cases, MRI-only navigation was used according to the surgeon’s discretion.

Preoperative MRI datasets were obtained from both in-house and external imaging facilities using 1.5 T or 3 T scanners (predominantly Siemens and Philips systems) with isotropic voxel spacing of approximately 1 mm^3^. Standard tumour protocols including T1-weighted sequences before and after contrast, T2-weighted, and FLAIR imaging were used whenever available.

High-resolution cranial CT scans, when performed, were acquired at our institution on a stationary 64-slice scanner (Somatom Definition AS, Siemens Healthineers, Germany) with a slice thickness of 0.6 mm.

Image fusion was performed using the automatic normalized-mutual-information algorithm implemented in the Brainlab Cranial 3.2 module, followed by manual verification and correction by the operating neurosurgeon.

### Frameless biopsy using the varioguide system

All intracranial biopsies in our department are routinely performed under general anaesthesia with the patient’s head fixed in a Mayfield clamp. Trajectory planning is, as a matter of internal standard, based on preoperative MRI and transferred to the neuronavigation system (Brainlab VarioGuide®, Brainlab AG, Munich, Germany). The biopsy path is defined to minimise tissue trauma and avoid critical structures. Arteries and veins are visualised in reconstructed multiplanar images to reduce the risk of intracerebral haemorrhage. Trajectories can be interactively adjusted without the need for stereotactic recalculation.

After pointer-based surface registration using anatomical landmarks, the trajectory is aligned via a flexible mechanical arm integrated into the navigation system. A small-diameter burr hole (Ø ≈ 2,5 mm) is created directly along the planned trajectory Biopsy specimens were obtained at the target site using a disposable, side-cutting Sedan biopsy needle (outer diameter 2.1 mm), compatible with the Brainlab VarioGuide® system. As the needle is withdrawn, light digital occlusion at the hub allows passive air entry into the tract, typically resulting in an intralesional air cavity of approximately 0.2–0.5 ml, sufficient for postoperative CT visualisation.

Routine postoperative CT is acquired approximately six hours after the procedure to exclude haemorrhagic complications and to visualise the air-filled biopsy tract for accuracy analysis.

### Data collection

**Fig. 1:**
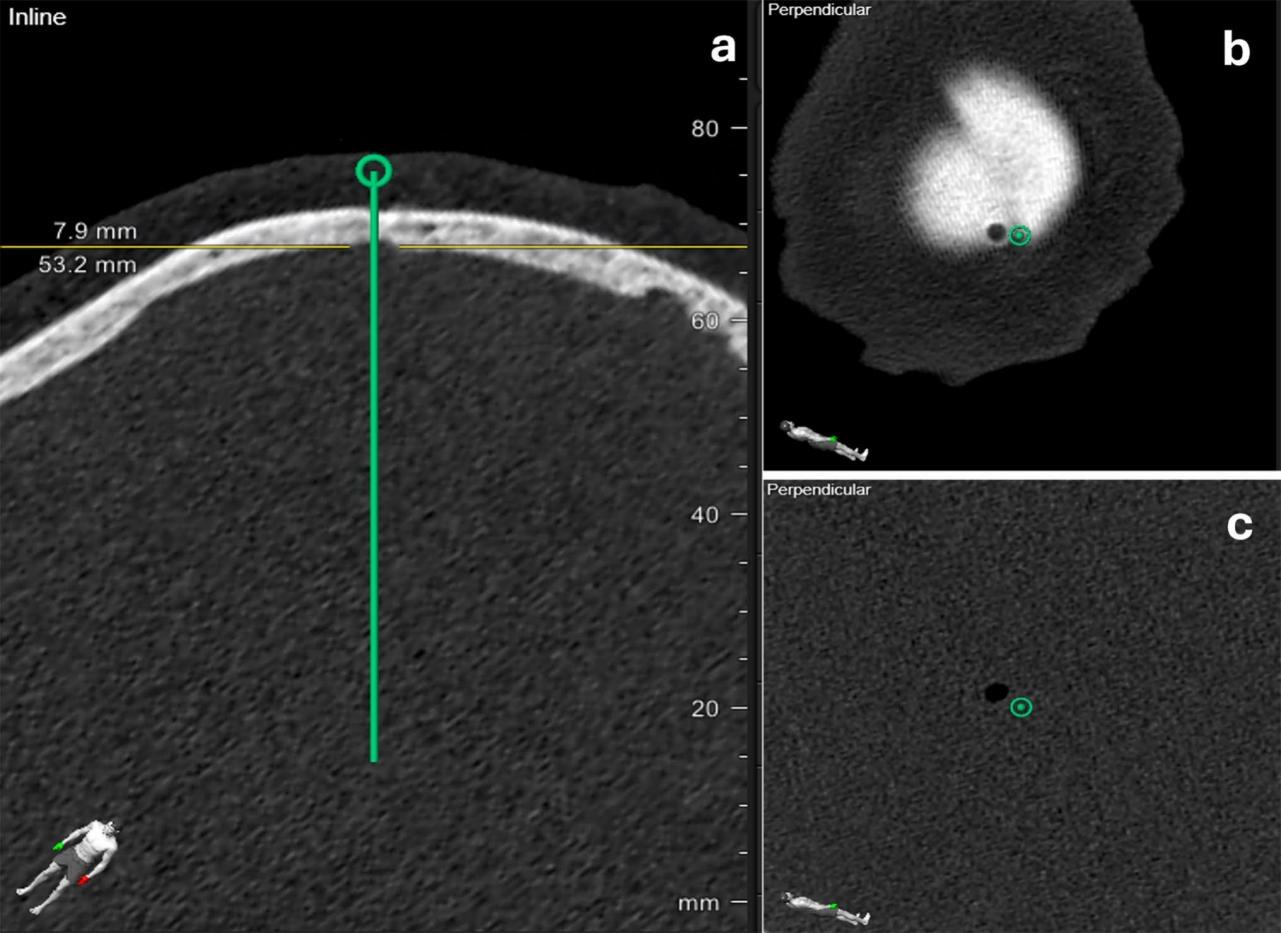
Postoperative CT scan demonstrating the inline (**a**) and perpendicular (**b**, **c**) views of the planned biopsy trajectory. In the linear view (**a**), the distance from the internal table of the skull to the planned target point was measured to assess target depth (53.2 mm). In the orthogonal views (**b**, **c**), deviations between the planned entry point and the actual burr hole, as well as between the planned target point and the actual biopsy cavity (air bubble), were evaluated to determine stereotactic accuracy

Accuracy was assessed using postoperative CT scans with multiplanar trajectory reconstructions. Entry point deviation was defined as the distance between the planned skull entry point and the centre of the actual burr hole as seen in Fig. 1b.

Target point deviation was measured as the distance between the planned target and the centre of the air-filled biopsy cavity as seen in Fig. 1c.

Measurements were performed in millimetres using trajectory-aligned CT reconstructions in two orthogonal planes: one parallel (inline) and one perpendicular to the planned needle path, as seen in Fig. 1.

When measuring the deviation of the target point, displacement in depth was not considered, as samples are often taken at multiple levels.

Additionally, the location and volume of the targeted lesion, the depth of the target point from the internal table of calvarium in millimetres, and intra- and postoperative complications were assessed.

**Fig. 2:**
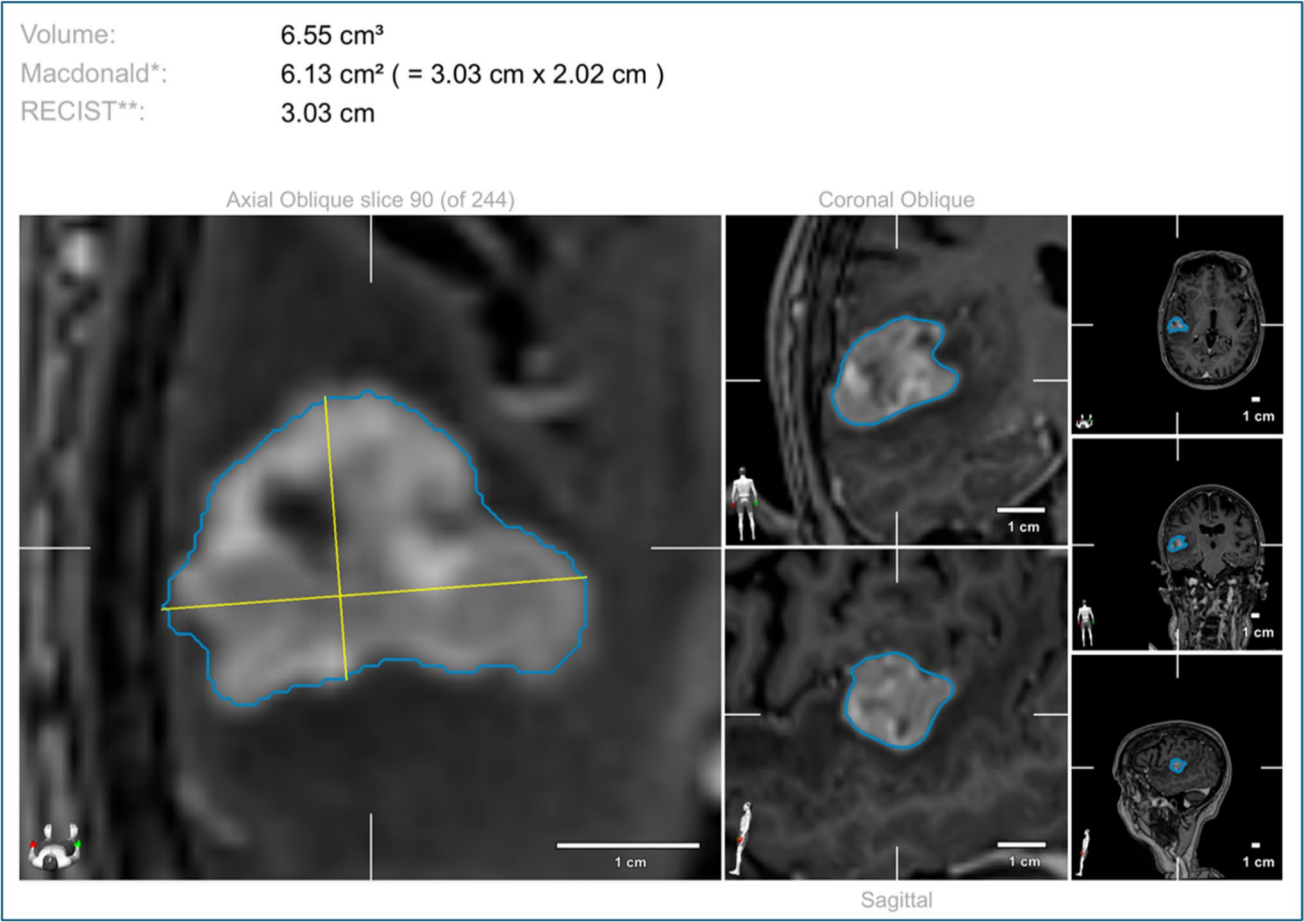
Volumetric analysis of a contrast-enhancing tumour using the SmartBrush tool in the Brainlab® navigation system. The lesion is segmented across axial, coronal, and sagittal planes, with the resulting 3D tumour volume calculated at 6.55 cm^3^. Bidimensional tumour size is presented according to the Macdonald (6.13 cm^2^, product of the two largest perpendicular diameters) and Response Evaluation Criteria in Solid Tumors (RECIST; 3.03 cm, longest single diameter) criteria. Tumour boundaries are highlighted in white, and orthogonal diameters are indicated by dashed lines

The volumetric analysis of the targeted lesion was conducted using the SmartBrush tool within the Brainlab system (Brainlab AG, Munich, Germany) as presented in Fig. 2.

### Statistical analysis

Frameless biopsies performed using MRI-only navigation were compared to those using MRI-CT-fusion based navigation, with a primary focus on targeting accuracy.

In addition, the influence of target volume and target depth on targeting deviation was assessed.

Statistical analyses were conducted using IBM SPSS Statistics (Version 30).

Unpaired t-tests were used to compare continuous variables between groups, and Fisher’s exact test was applied for categorical variables with small sample sizes.

Pearson’s correlation coefficient was calculated to assess the relationship between continuous parameters (e.g., target volume or depth and deviation).

A *p*-value below 0.05 was considered statistically significant.

Given the limited size of the MRI-CT fusion subgroup (*n* = 18), multivariate modelling was not applied to avoid overfitting and unstable estimates. Exploratory univariate comparisons and correlation analyses were therefore performed.

## Results

A total of 113 patients who underwent frameless stereotactic procedures using the VarioGuide® system were initially identified.

Fourteen patients were excluded from the analysis for the following reasons: absence of postoperative CT imaging (*n* = 5), conversion to frame-based stereotactic biopsy (*n* = 2), or execution of a different procedure than tumour biopsy, such as navigated abscess drainage (*n* = 7).

Consequently, 99 patients who underwent either an MRI-only or an MRI-CT fusion–based frameless stereotactic tumour biopsy were included in the final analysis.

Of these, 81 cases (81.8%) were performed using MRI-only navigation, and 18 cases (18.2%) using MRI-CT fusion–based navigation. The cohort comprised 55 females and 44 males, with a balanced gender distribution between groups. The time from MRI acquisition to biopsy did not exceed ten days in any case (median = 6 days).

Lesion localisation varied, with the majority located in the frontal lobe (*n* = 43), followed by the temporal lobe (*n* = 21), parietal lobe (*n* = 15), periventricular region (*n* = 13), basal ganglia (*n* = 11), occipital lobe (*n* = 4), cerebellum (*n* = 2), and insular region (*n* = 2).

**Fig. 3:**
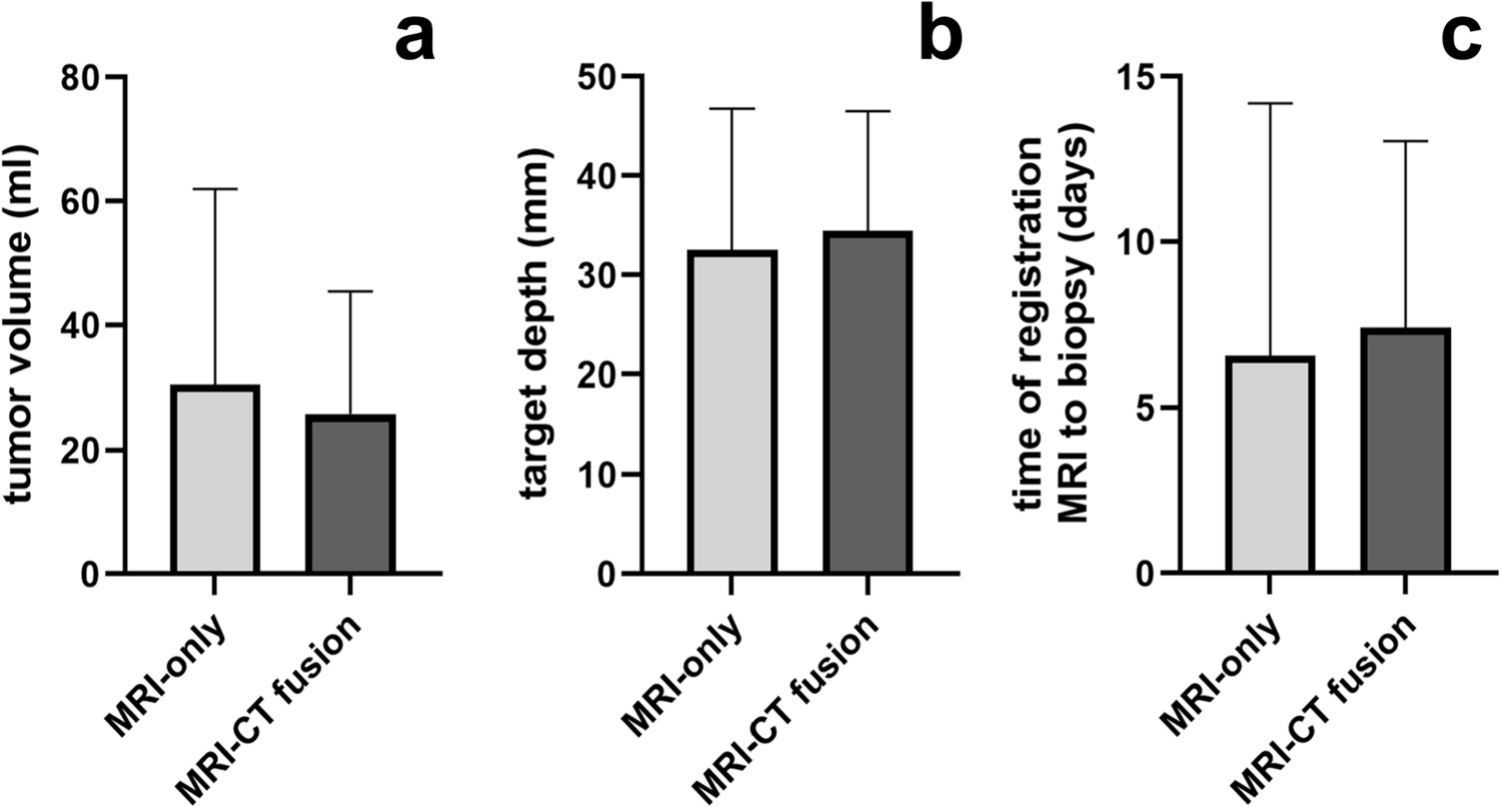
Comparison of tumour volume, target depth, and registration-to-biopsy interval between CT-based (MRI-CT-fusion based) and MRI-only navigated frameless biopsies. **A** Tumour volumes did not differ significantly between groups (CT-based: 25.8 ± 19.7 ml vs. MRI-only: 30.6 ± 31.4 ml; *p* = 0.54). **B** The mean distance from the internal table of the skull to the target point was comparable between groups (CT-based: 34.5 ± 12.0 mm vs. MRI-only: 32.5 ± 14.3 mm; *p* = 0.59). **C** The time from MRI registration to biopsy was also similar (CT-based: 7.4 ± 5.9 days vs. MRI-only: 6.5 ± 7.7 days; *p* = 0.68)

The time from MRI registration to biopsy did not differ significantly between groups (MRI-only: 6.5 ± 7.7 days vs. MRI-CT fusion: 7.4 ± 5.9 days; *p* = 0.68), as shown in Fig. 3C.

Similarly, no significant differences were found in target lesion volume (MRI-only: 30.6 ± 31.4 ml vs. MRI-CT fusion: 25.8 ± 19.7 ml; *p* = 0.54) or in the distance from entry point to target (MRI-only: 32.5 ± 14.3 mm vs. MRI-CT fusion: 34.5 ± 12.0 mm; *p* = 0.59), as presented in Fig. 3A and 3B.

The total procedural duration did not differ significantly between groups (MRI-only: 68.3 ± 19.6 min vs. MRI-CT fusion: 73.2 ± 25.7 min; *p* = 0.37).

Incision-to-suture time was slightly longer in the MRI–CT fusion group (25.4 ± 22.2 min) compared to the MRI-only group (18.7 ± 9.2 min, p = 0.04).

“Incision-to-suture time” is defined as pure surgical duration between skin incision and closure, whereas “total OR time” denotes the total room occupancy time from patient entry to exit, including positioning, sterile preparation, surgery, and wound closure.

**Fig. 4:**
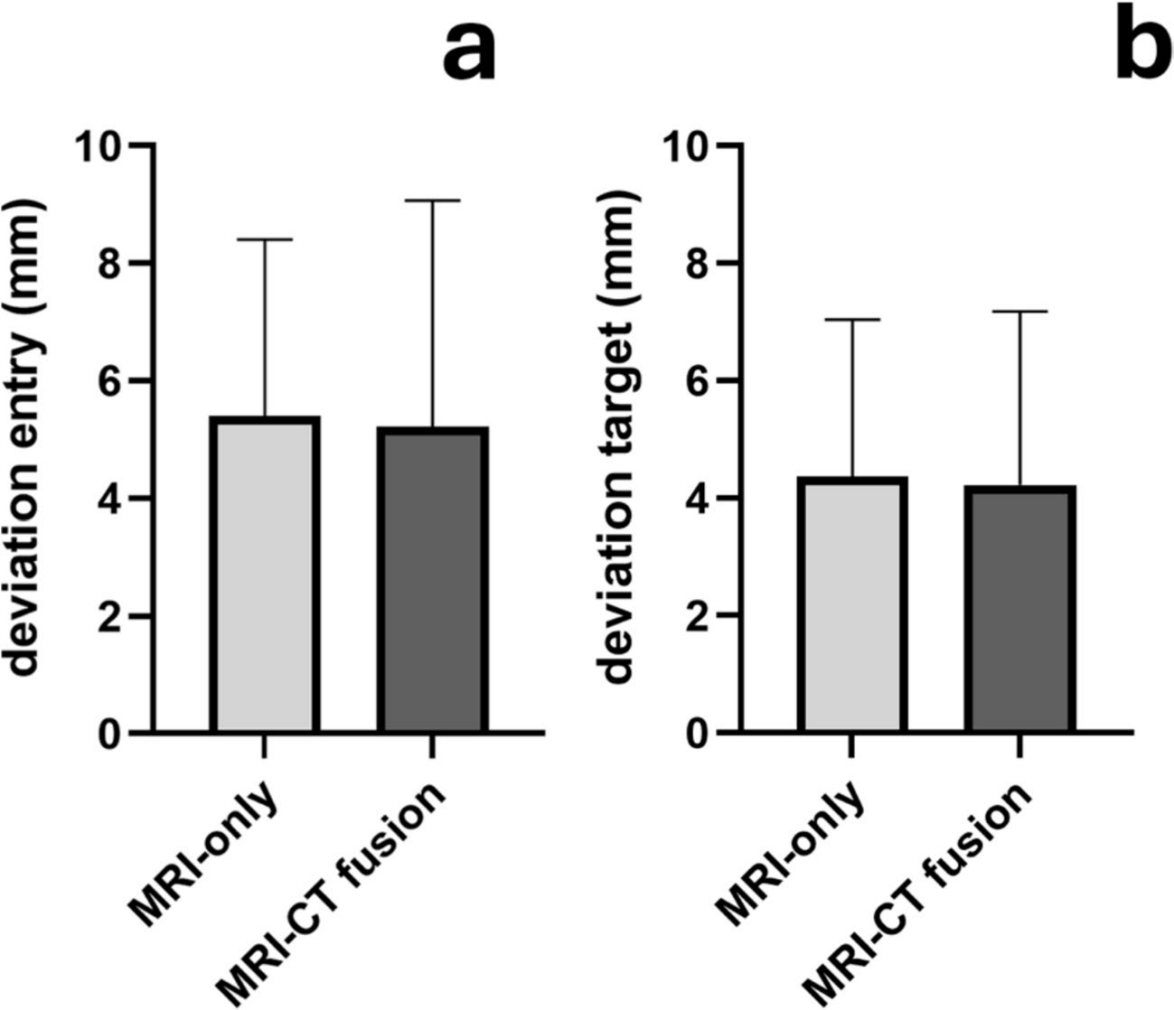
Observed deviation at the entry point (**A**) and at the target point (**B**) in CT-based ((MRI-CT-fusion based) and MRI-only navigated frameless stereotactic biopsies. Mean deviations were similar between groups both at the entry (CT-based: 5.2 ± 3.9 mm; MRI-only: 5.4 ± 3.0 mm; *p* = 0.84) and at the target (CT-based: 4.2 ± 3.0 mm; MRI-only: 4.4 ± 2.7 mm; *p *= 0.85), indicating comparable accuracy across techniques

Accuracy, assessed by deviations at the entry and target points, was comparable between both groups. The mean entry point deviation was 5.2 ± 3.9 mm in the MRI-CT-fusion based group and 5.4 ± 3.0 mm in the MRI-only group (*p* = 0.84; Fig. 4A). The mean deviation at the target point was 4.2 ± 3.0 mm in the MRI-CT-fusion based group and 4.4 ± 2.7 mm in the MRI-only group (*p* = 0.85; Fig. 4B).

**Fig. 5:**
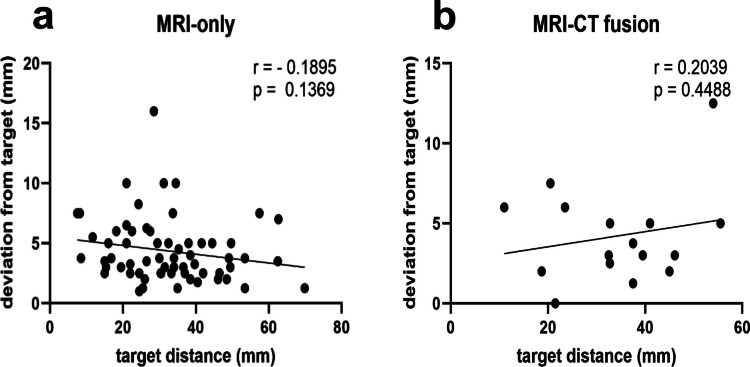
Correlation analysis between target depth (measured as distance from the inner table) and deviation from the planned biopsy target in frameless stereotactic procedures. **A** MRI-only group: no significant correlation was observed between target distance and deviation (*r* = –0.1895, *p* = 0.1369). **B** MRI-CT fusion group: no significant correlation between target distance and deviation (*r* = 0.2039, *p* = 0.4488). All correlation coefficients were calculated using Pearson’s correlation analysis

Regarding target depth (measured from the inner table), no significant correlations with target deviation were found in either group (MRI-CT-fusion-based: *r* = 0.2039, *p* = 0.4488; MRI-only: *r* = –0.1895, *p* = 0.1369; Fig. 5C and 5D).

Complication rates were low in both groups. A single severe postoperative haemorrhage occurred in the MRI-only group (1.2%) and led to major clinical deterioration and switch to palliative care. No such events were observed in the MRI-CT fusion group. The single severe postoperative haemorrhage occurred in a patient with a highly malignant lesion and presumably markedly altered vascular architecture. Given the multifactorial nature of such events, this complication was not interpreted as being related to the navigation modality itself, but in our opinion, rather reflected the inherent procedural risk of stereotactic biopsy in vulnerable tumour tissue.

Additional intraoperative or diagnostic deviations from standard workflow were recorded but not classified as complications. These included one intraoperative awakening requiring re-navigation in the MRI-only group, as well as one aborted procedure with conversion to craniotomy due to insufficient navigational reliability during the procedure, and one technical mispuncture in the MRI-CT fusion group.

In seven patients (7.1%), no reliable histopathological diagnosis could be established (MRI-only: *n* = 5; MRI-CT fusion: *n *= 2).

An overview of patient characteristics, lesion localisation, procedural timing, and all recorded events is provided in Table 1.

**Table 1:** Comparison of demographic, procedural, accuracy-related, and complication parameters between MRI-CT-fusion based and MRI-only navigated frameless stereotactic biopsies

Parameter	MRI-only (*n* = 81)	MRI-CT-fusion based (*n* = 18)	*p*-value
Number of procedures (%)	81 (81.8%)	18 (18.2%)	-
Sex			
Female	46	9	n.s. (0.61)
Male	35	9	n.s. (0.61)
Localization			
Frontal lobe	33	10	n.s. (0.30)
Parietal lobe	14	1	n.s. (0.29)
Temporal lobe	17	4	n.s. (0.99)
Occipital lobe	4	0	n.s. (0.99)
Basal ganglia	10	1	n.s. (0.68)
Cerebellum	2	0	n.s. (0.99)
Periventricular region	12	1	n.s. (0.45)
Insular	2	0	n.s. (0.99)
Timing			
Days from CT to biopsy (mean ± SD, range)	n/a	2.1 ± 2.4 (0–9)	-
Days from MRI to biopsy (mean ± SD, range)	6.5 ± 7.7 (0–35)	7.4 ± 5.9 (1–18)	n.s. (0.68)
Target characteristics			
Target volume (ml, mean ± SD, range)	30.6 ± 31.4 (0.5–160.2)	25.8 ± 19.7 (3.0–58.4)	n.s. (0.54)
Target distance from inner table (mm, mean ± SD, range)	32.5 ± 14.3 (7.5–69.9)	34.5 ± 12.0 (11.0–55.5)	n.s. (0.59)
Operative parameters			
Incision-to-suture time = pure surgical duration; (min, mean ± SD, range)	18.7 ± 9.2 (4–50)	25.4 ± 22.2 (11–102)	* (0.04)
Total operative time = surgical workflow including draping and dressing (min, mean ± SD, range)	68.3 ± 19.6 (27–115)	73.2 ± 25.7 (34–152)	n.s. (0.37)
Total OR time = time from patient entry to exit from operating room. (min, mean ± SD, range)	211.4 ± 170.1 (105–152)	163.8 ± 42.0 (105–272)	n.s. (0.26)
Accuracy			
Entry point deviation (mm, mean ± SD, range)	5.4 ± 3.0 (1.0–16.5) (n = 76)	5.2 ± 3.9 (0.0–15.0) (n = 15)	n.s. (0.84)
Target point deviation (mm, mean ± SD, range)	4.4 ± 2.7 (1.0–16.0) (n = 63)	4.2 ± 3.0 (0–12.5) (n = 16)	n.s. (0.85)
Complications			
Severe postoperative hemorrhage	1 (1,2%)	0 (0.0%)	n.s. (0.99)
Other intra-operative or diagnostic events			
Aborted procedure or conversion to craniotomy	0 (0.0%)	1 (5,6%)	n.s. (0.18)
No reliable histo-logical diagnosis	5 (6,2%)	2 (11,1%)	n.s. (0.61)
Intraoperative awakening and re-navigation	1 (1,2%)	0 (0,0%)	n.s. (0.99)

Values are reported as mean ± standard deviation (range), unless otherwise indicated

Statistical comparisons were performed using unpaired t-tests for continuous variables and Fisher’s exact test for categorical variables. *Indicates statistically significant differences (*p* < 0.05); n.s. = not significant

## Discussion

Our study provides updated insights into the clinical performance of frameless stereotactic brain biopsy techniques under routine conditions. Although not designed as a comparative trial, the retrospective nature of our data allowed a descriptive comparison between biopsies performed using MRI-only versus MRI-CT fusion–based navigation.

A total of 99 frameless biopsies were analysed, of which 18 (18.2%) utilised MRI–CT fusion and 81 (81.8%) relied on MRI-only navigation. Overall, the procedures showed comparable accuracy without significant difference in targeting deviation between both approaches. Entry point deviations (5.2 ± 3.9 mm vs. 5.4 ± 3.0 mm) and target point deviations (4.2 ± 3.0 mm vs. 4.4 ± 2.7 mm) did not differ significantly, confirming that frameless stereotactic biopsy provides precise targeting regardless of the image-guided navigation strategy used for registration.

These findings are consistent with previous reports indicating similar accuracy levels across different frameless systems and planning modalities [8, 14, 16].

This interpretation is supported by findings from Gocmen et al., who investigated frameless biopsy accuracy in a dual-centre cohort of 88 patients, including eloquent and small lesions [8]. Their results indicate that MRI-CT fusion can reduce targeting deviation in cases where standalone MRI-based navigation is limited by image distortion or reduced contrast resolution. However, as in our cohort, no systematic superiority was demonstrated for MRI-CT fusion in general use.

While the mean targeting deviation of approximately 4–5 mm is within the expected range for frameless stereotactic systems, it remains geometrically relevant, particularly for deep or eloquent targets. Even small angular deviations can translate into several millimetres of error at larger depths, potentially affecting diagnostic yield and safety. However, smaller thalamic or brainstem lesions — where such deviations would have the greatest impact — were not part of the present cohort, as these are routinely approached with frame-based techniques at our institution. Consequently, the applicability of our findings to deep or highly eloquent targets is limited, and the results should be interpreted within this clinical context.

In our cohort, total procedural time did not differ significantly between groups, suggesting that MRI-CT fusion integration does not meaningfully prolong the surgical workflow. The slightly longer incision-to-suture time observed in the MRI–CT fusion group is unlikely to reflect a modality-related effect, as image fusion was performed preoperatively.

Instead, it may represent variability in operator experience or sampling strategy, since some procedures were performed by attending surgeons and others by residents under supervision.

These parameters were not controlled for in this retrospective analysis, and future studies should account for surgeon experience to better isolate the impact of the navigation approach.

MRI-CT fusion–based frameless biopsy may serve as a valuable extension of conventional MRI-only navigation in selected clinical scenarios—such as when MRI quality is compromised by motion artefacts, implants, or poor soft-tissue contrast, or when high-resolution cranial CT data are already available. While CT-based registration introduces additional procedural steps, cost and a moderate increase in radiation dose—typically within the range of a standard diagnostic cranial CT— it may obviate the need for repeat MRI and facilitate trajectory planning in complex anatomical settings or resource-limited environments.

Our data highlight the comparable reliability of both approaches and contribute to a more nuanced understanding of their respective roles in routine neurosurgical practice. Ultimately, the choice of navigation strategy should be guided by clinical context, lesion characteristics, and imaging quality.

Future studies employing a case–control or matched-cohort design—with comparable lesion characteristics, imaging quality, and anatomical location—would substantially strengthen the validity of inter-modality comparisons and help isolate the specific contribution of CT integration.

### Limitations

This study has several limitations inherent to its retrospective design. The relatively small number of patients in the MRI-CT fusion group (*n* = 18) limits statistical power and may have masked subtle differences in targeting accuracy or complication rates. Group assignment was not randomised, and selection bias cannot be excluded. As this was a retrospective analysis of routine clinical practice, allocation to MRI-only or MRI–CT fusion navigation was not based on predefined criteria but reflected the availability of suitable imaging data and individual surgeon preference. This non-standardised approach may have introduced selection bias and potential confounding between groups. Furthermore, image fusion quality and registration accuracy were not independently validated, and postoperative accuracy measurements relied on the localisation of air cavities, which may introduce minor variability. Additionally, small angular deviations in VarioGuide arm positioning (typically in the range of 0.1° to 0.3°) were pragmatically tolerated in line with standard clinical workflow. This approach may not reflect the stricter alignment protocols that would be applied in prospective studies focused specifically on navigational accuracy. Future investigations should incorporate standardised calibration procedures to minimise such variation and enable more robust assessment of targeting precision.

In addition, no multivariate analysis was performed because of the limited sample size, particularly within the MRI–CT fusion subgroup (*n* = 18). Applying multivariate models under these conditions would have led to model overfitting and unstable estimates. For this reason, only exploratory univariate comparisons were conducted, which limits the statistical power to detect subtle interactions between variables.

Prospective, standardised trials with larger and balanced cohorts are needed to confirm findings and to better define patient subgroups who may benefit from CT integration.

## Conclusion

Frameless stereotactic biopsy remains a cornerstone in the diagnostic workup of intracranial lesions. Our data supports the safety and precision of this technique under routine clinical conditions, with no statistically significant difference observed between MRI-only and MRI–CT fusion–based navigation. Based on the targeting accuracy reported in our cohort (mean deviation approximately 5 mm), frameless stereotactic biopsies using the VarioGuide system appear most suitable for lesions ≥ 9–10 mm in diameter, where the probability of obtaining diagnostic tissue is sufficiently high. For smaller targets, frame-based systems may offer enhanced reliability.

Both MRI-only and MRI–CT fusion navigation can be safely applied in routine clinical practice; however, given the retrospective design, group imbalance, and lack of matching, the present results should be regarded as descriptive and hypothesis-generating rather than confirmatory. Future prospective or matched-cohort studies are warranted to validate these findings and to define specific scenarios in which CT integration may provide a measurable advantage.

## Data Availability

The datasets generated and analysed during the current study are available from the corresponding author on reasonable request.

## Declarations

### Reporting guideline

The manuscript was prepared in accordance with the STROBE (Strengthening the Reporting of Observational Studies in Epidemiology) statement [20].
